# WGSQuikr: Fast Whole-Genome Shotgun Metagenomic Classification

**DOI:** 10.1371/journal.pone.0091784

**Published:** 2014-03-13

**Authors:** David Koslicki, Simon Foucart, Gail Rosen

**Affiliations:** 1 Mathematics Department, Oregon State University, Corvallis, Oregon, United States of America; 2 Department of Mathematics, University of Georgia, Athens, Georgia, United States of America; 3 Department of Electrical and Computer Engineering, Drexel University, Philadelphia, Pennsylvania, United States of America; Auburn University, United States of America

## Abstract

With the decrease in cost and increase in output of whole-genome shotgun technologies, many metagenomic studies are utilizing this approach in lieu of the more traditional 16S rRNA amplicon technique. Due to the large number of relatively short reads output from whole-genome shotgun technologies, there is a need for fast and accurate short-read OTU classifiers. While there are relatively fast and accurate algorithms available, such as MetaPhlAn, MetaPhyler, PhyloPythiaS, and PhymmBL, these algorithms still classify samples in a read-by-read fashion and so execution times can range from hours to days on large datasets. We introduce WGSQuikr, a reconstruction method which can compute a vector of taxonomic assignments and their proportions in the sample with remarkable speed and accuracy. We demonstrate on simulated data that WGSQuikr is typically more accurate and up to an order of magnitude faster than the aforementioned classification algorithms. We also verify the utility of WGSQuikr on real biological data in the form of a mock community. WGSQuikr is a Whole-Genome Shotgun QUadratic, Iterative, 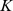-mer based Reconstruction method which extends the previously introduced 16S rRNA-based algorithm Quikr. A MATLAB implementation of WGSQuikr is available at: http://sourceforge.net/projects/wgsquikr.

## Introduction

While 16S rRNA amplicon sequencing is a popular approach to reconstructing the taxonomic composition of a bacterial community, there are some limitations to this approach. For example, multiple copies of 16S rRNA genes in a single organism and nearly identical 16S rRNA genes in other species can both lead to mis-estimates of bacterial compositions [1]. These and other considerations have contributed to an increased usage of whole-genome shotgun (WGS) sequencing to analyze microbial communities. However, the large amount of short reads resulting from WGS methods (ranging from 70 million 200 bp-length reads for Ion Torrent's Proton Torrent sequencer, to 3 billion 100 bp-length reads for Illumina's HiSeq, to 15 million 36 bp-length reads for Illumina's MiSeq) necessitates fast and accurate algorithms to process these large amounts of data. Current methods, while relatively accurate, can still take from 8 hours (MetaPhyler [2]) to 4 days (PhymmBL [3]) to analyze a relatively small dataset of 70 thousand 300 bp reads [2].

We introduce a method that extends the previously introduced 16SrRNA-based algorithm Quikr [4], allowing for the accurate analysis of very large whole-genome shotgun datasets (billions of reads) on a laptop computer in under an hour. This is facilitated by leveraging ideas from compressive sensing to reconstruct all taxonomic relative abundances of a bacterial community simultaneously (as opposed to read-by-read classification). Beyond significant speed improvements, we demonstrate on simulated data that this method has, on average, better reconstruction fidelity than any other technique to date, even down to the genus level.

Briefly, our method first measures the frequency of 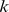-mers (for a fixed 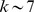) in a database of known bacterial genomes, calculates the frequency of 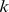-mers in a given sample, and then reconstructs the concentrations of the bacteria in the sample by solving a system of linear equations under a sparsity assumption. To solve this system, we employ MATLAB's [5] iterative implementation of nonnegative least squares and hence we refer to this method as *WGSQuikr*: Whole-Genome Shotgun QUadratic, Iterative, 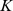-mer based Reconstruction. We point out that WGSQuikr has not yet been optimized for performance but still demonstrates a significant speed improvement over existing methods.

## Methods

### 2.1. 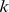-mer Training Matrix

The training step consists of converting an input database of whole bacterial genomic sequences (with their associated plasmid sequences) into a 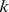 *-mer training matrix*. For a fixed 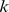-mer size, we calculate the frequency of each 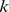-mer in each database sequence. Hence, given a database of genome sequences 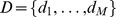, the 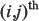 entry of the 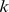-mer training matrix 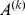 is the frequency of the 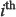 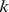-mer (in lexicographic order) in the 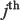 sequence 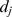.

Herein, we consider a single, manually curated database 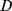 consisting of 1,401 bacterial genomes and 1,082 plasmids, resulting in 2,483 unique sequences, which along with their taxonomic information, were retrieved from NCBI [6] in October, 2012. The bacterial sequences in this database cover 1,109 species and 614 genera.

### 2.2. Sample 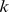-mer Frequencies

Given a sample dataset of WGS reads, we orient all the reads in the forward direction, and then calculate the frequency of all 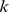-mers in the entire sample. We refer to this vector 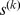 as the *sample* 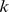 *-mer frequency vector*.

### 2.3. Sparsity Promoting Quadratic Optimization

We assume that the given environmental sample only contains bacteria that exist in the database 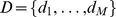 being utilized. Hence we can represent the composition of the sample as a vector 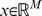 with nonnegative entries summing to one (i.e. a probability vector) where 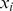 is the concentration of the organism with genome 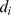. However, as will be demonstrated in subsection 3.10, WGSQuikr still performs adequately when the sample *does* contain novel bacteria not in the database being utilized.

The problem at hand is then to reconstruct the bacterial concentrations 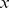 by solving the linear system (2.1)
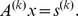



Equation (2.1) is solved by using a sparsity-promoting optimization procedure motivated by techniques used in the compressive sensing literature. Sparsity is emphasized since it is reasonable to assume that relatively few bacteria from the database 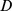 are actually present in the given sample. We use a variant of nonnegative basis pursuit denoising [7], [8] which reduces to a nonnegative least squares problem. Unlike the 16S rRNA version of Quikr [4], WGSQuikr experiences no convergence issues thanks to the inclusion of an adaptive choice of a regularization parameter which is calculated individually for each dataset. The details regarding this procedure are contained in Appendix S1.

### 2.4. Reconstruction Metrics

We denote the *actual* and *predicted* concentrations of the bacteria as probability vectors 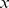 and 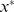 respectively. The reconstruction metric primarily employed herein is the 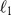 distance between 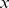 and 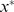: 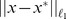. This quantity takes values between 0 and 2 (with perfect reconstruction being 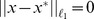) and is commonly referred to as “total error” (as it is the total of the absolute errors). The term *reconstruction fidelity* will be used to communicate generically how well 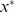 approximates 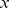. We will mainly be concerned with reconstruction fidelity down to the genus level since the assumption given in subsection 2.3 indicates that WGSQuikr is applicable in situations where the given metagenomic sample does not contain (too distantly related) novel taxonomic units absent from the training database. This is more likely to be the case at the genus level than at the species or strain level.

### 2.5. Simulated Data

To test the performance of the WGSQuikr method, the shotgun read simulator Grinder [9] was used to generate 720 simulated WGS datasets totaling over 1 billion reads. These datasets have a wide range of differing characteristics designed to replicate a range of technologies in a variety of conditions (for example: differing species abundances, read coverages, read lengths, error models, abundance models, etc.). The particular parameter values can be found in Appendix S1. We verified that our results do not depend on the randomly chosen bacterial species in each dataset by re-running each simulation 5 times and observing that the results in section 3.6 do not change.

### 2.6. Mock Communities

To benchmark the Quikr method on real biological data, we examined the “even” mock microbial community (NCBI SRR172902) developed by the Human Microbiome Project [10]. This community contains known concentrations of bacteria from 21 different organisms that span a diverse range of properties (GC content, genome size, etc.).

## Results

There are many whole-genome shotgun metagenomic classifiers that WGSQuikr can be compared to. A selection includes NBC [11], [12], Phymm [3], PhymmBl [3], [13], MetaPhyler [2], RITA [14], PhyloPythiaS [15], MetaPhlAn [16], Genometa [17] and MetaID [18].Typically, these algorithms classify a sample in a read-by-read fashion against a known database. Briefly, NBC accomplishes this in a Bayesian framework utilizing 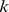-mer counts. Phymm and PhymmBL use interpolated Markov models to characterize variable-length oligonucleotides. MetaPhyler and MetaPhlAn use clade-identifying marker genes. Genometa and RITA are BLAST-based techniques, and MetaID uses large common and unique 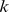-mers to classify reads. It has been shown [2], [16], [17] that the methods roughly rank in terms of increasing execution time as: MetaPhlAn, MetaPhyler, PhyloPythiaS, Phymm, PhymmBL, NBC, Genometa, and RITA (MetaID [18] details no run-time data).

WGSQuikr differs from all of these methods as it classifies an entire dataset simultaneously rather than in a read-by-read fashion. Furthermore, the other 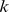-mer based techniques typically use 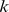-mers for 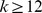, whereas WGSQuikr uses 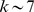. As WGSQuikr is intended to be used as a fast classification method at a taxonomic level in which few novel taxa appear, we choose to compare to the two fastest methods available: MetaPhlAn and MetaPhyler. WGSQuikr and these two algorithms will be evaluated on all simulated data and the mock community using the default parameters.

### 3.7. Speed Comparison

**Figure 1:**
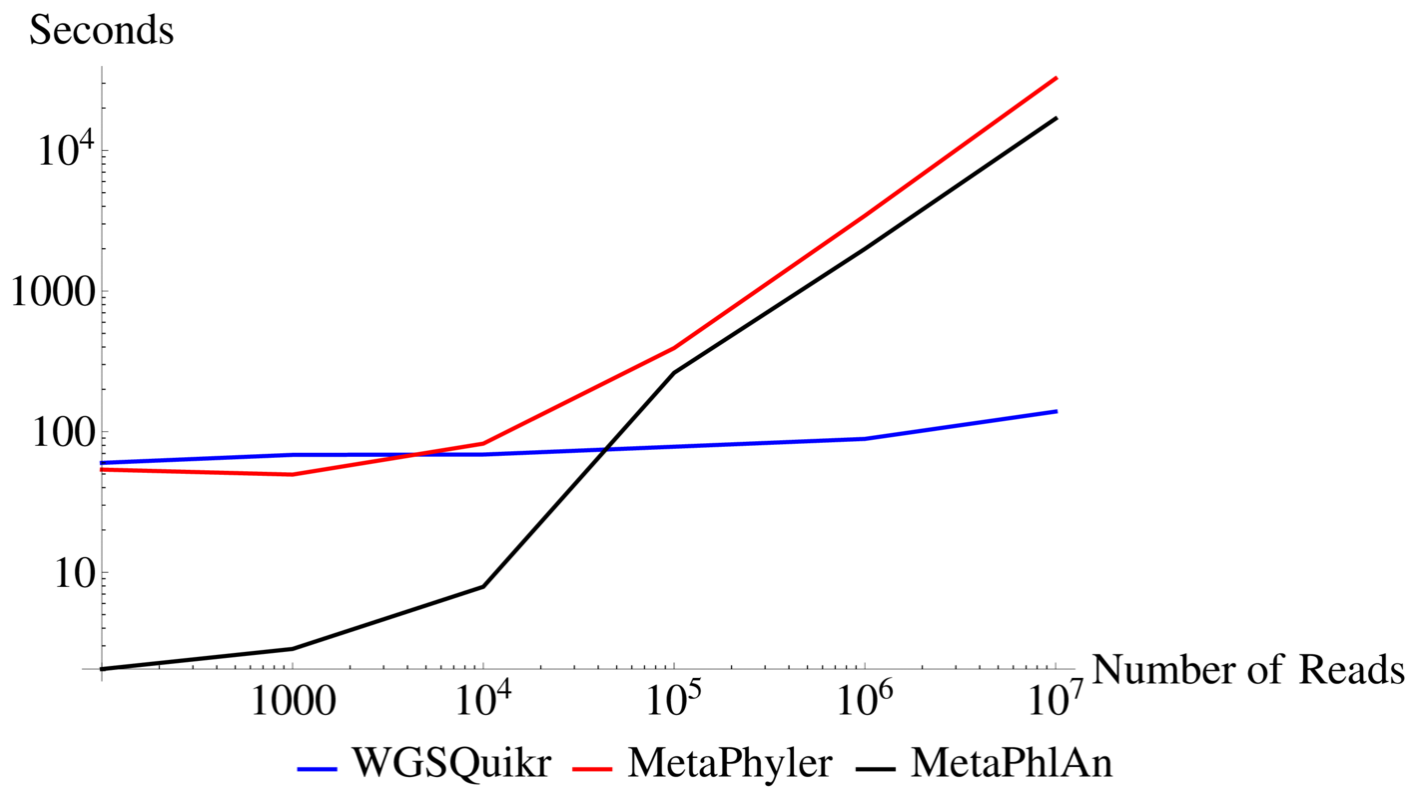
Log-log plot of number of reads versus execution time (seconds) for WGSQuikr, MetaPhyler and MetaPhlAn.

Throughout the following, we fixed the 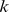-mer size at 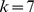. We observed the general trend that the algorithm execution time increased exponentially as a function of 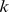, while the 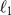-error decreased roughly linearly. We chose 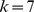 as this provided a reasonable tradeoff between fast execution time and low reconstruction error. Figure 1 shows a log-log plot of the execution time for WGSQuikr, MetaPhyler, and MetaPhlAn on datasets ranging from 100 reads to 10 million reads of 75 bp in length. Figure 1 includes the time required to form the sample 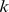-mer frequency vector for the WGSQuikr algorithm. As 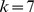 is relatively small, the time required to form this vector is negligible (e.g. for a sample with 1 M 75 bp reads, it takes less than 5 seconds to form the sample 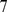-mer frequency vector). The execution time is nearly constant for WGSQuikr to solve (2.1) via the algorithm detailed in Appendix S1. This is due to the algorithm taking as input the 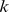-mer frequency vector, whose size depends only on 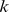, not the size of the given dataset. This also explains the reason for the significant speed improvement of WGSQuikr: the entire sample is classified simultaneously, as opposed to in a read-by-read fashion such as with MetaPhyler or MetaPhlAn.

**Figure 2:**
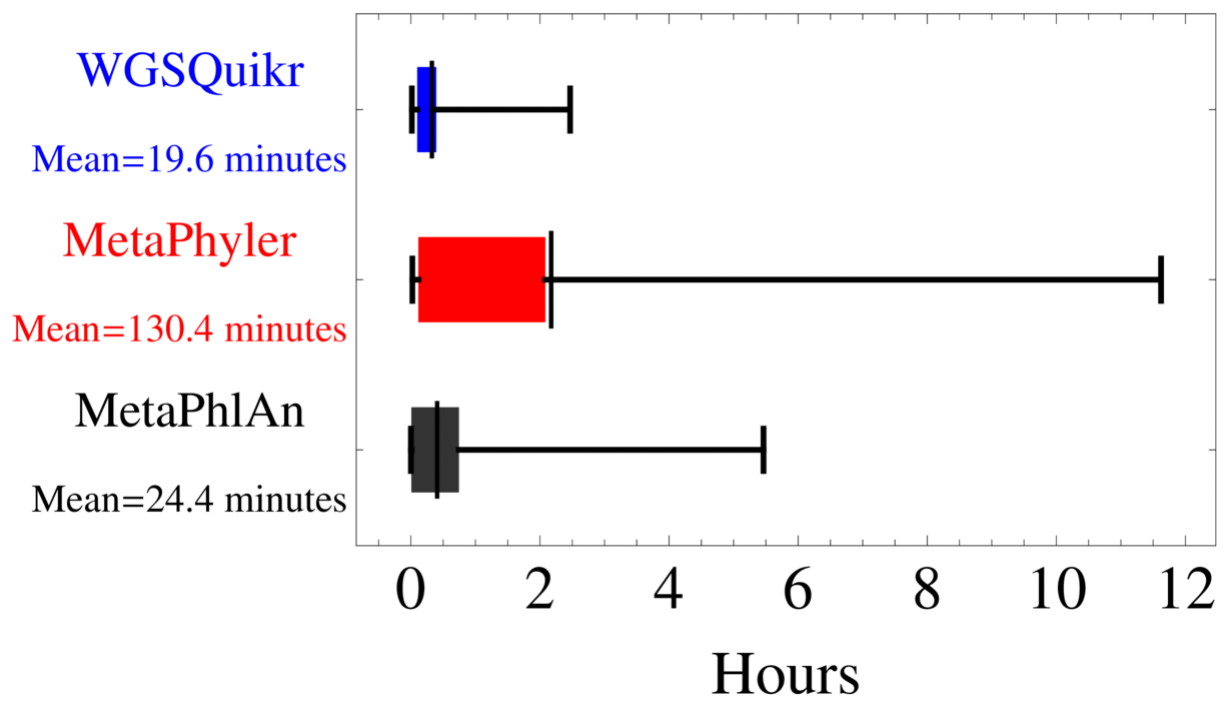
Box-and-whisker plot of execution time (in minutes) on the simulated experiments for WGSQuikr, MetaPhyler, and MetaPhlAn. The boxes demarcate 75% quantiles, whiskers demarcate range, and the vertical black bars are drawn at the mean.

Figure 2 shows a box-and-whisker plot of the execution time for WGSQuikr, MetaPhyler, and MetaPhlAn on the simulated datasets described in subsection 2.5. Note the significant improvement in speed: the average execution time of WGSQuikr is over 6 times faster than the average MetaPhyler execution time. For the larger datasets (5 M reads), WGSQuikr is on average 27 times faster than MetaPhyler and 5 times faster than MetaPhlAn.

### 3.8. Simulated Data Results

#### 3.8.1. Reconstruction Error

**Figure 3:**
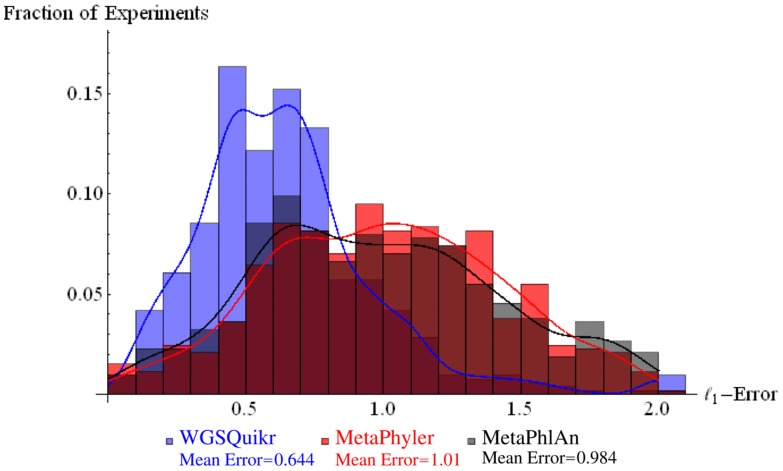
Histogram of 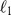-error versus fraction of simulated experiments at the genus level for WGSQuikr, MetaPhyler, and MetaPhlAn.

We evaluated the 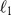-error at the genus level on the simulated datasets and summarize the mean 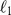-error at the genus level in table 1. The histogram in figure 3 shows the 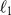-error versus fraction of the simulated datasets for WGSQuikr, MetaPhyler, and MetaPhlAn. Also included is a smooth kernel distribution approximation of each of the histograms (shown as lines in figure 3) to emphasize how WGSQuikr typically has less error than MetaPhyler and MetaPhlAn.

**Table 1:** Comparison of mean 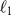-errors at the genus level (smaller values are better).

Method	Mean 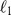 -error
WGSQuikr	0.644
MetaPhyler	1.006
MetaPhlAn	0.984

We hypothesize that the reason WGSQuikr demonstrates such an improvement in 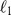-error over MetaPhyler and MetaPhlan is WGSQuikr's ability to very accurately reconstruct the frequency of the most abundant organisms in a sample. Indeed, at the genus level, the mean 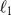-error decreased by 31% when focusing on only the top 10 most abundant genera. See subsection 3.9 for further supporting evidence.

#### 3.8.2. Reconstruction Fidelity vs Simulation Parameters

**Figure 4:**
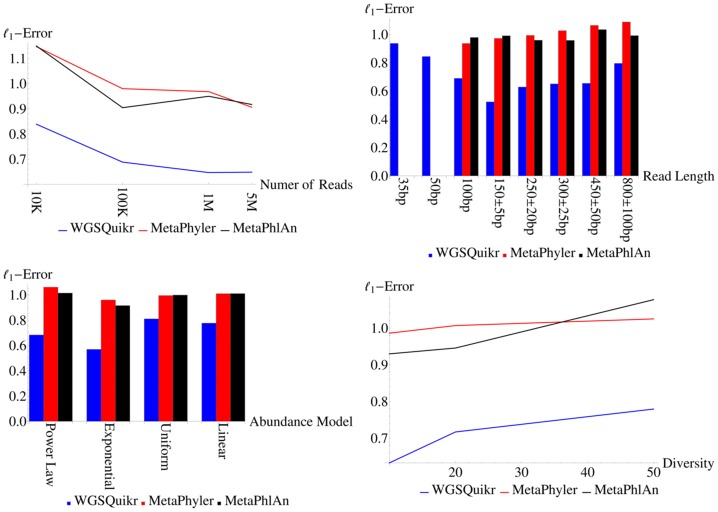
Mean 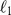-error at the genus level as a function of simulated dataset parameters for each method. MetaPhyler and MetaPhlAn failed to run on the datasets where reads were 35 bp or 50 bp in length.

In order to investigate what properties of a given dataset influence the reconstruction error of WGSQuikr, we grouped the simulated datasets by each simulation parameter (number of reads, read length, abundance model, or diversity). Figure 4 summarizes the mean error of WGSQuikr as a function of each one of these parameters, and includes the results for MetaPhyler and MetaPhlAn for comparison.

It is interesting to note that WGSQuikr runs particularly well on short read data. Indeed, WGSQuikr gave reasonable results when the read length was as short as 35 bp or 50 bp long, whereas MetaPhyler and MetaPhlAn both failed to return results in such cases. Furthermore, WGSQuikr exhibits roughly half as much 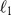-error (0.52) as MetaPhyler (0.97) and MetaPhlAn (0.99) for datasets consisting of reads normally distributed around 150 bp.

Given a larger number of reads, a lower diversity, and an abundance model closer to exponential, all three methods experienced improvement in reconstruction fidelity. Interestingly, longer read lengths seemed to negatively impact all three methods.

### 3.9. Mock Community Results

To show that WGSQuikr can allow for fast, high-level analysis of large datasets on a laptop computer, we analyzed the mock community described in subsection 2.6 using a 2013 Macbook Air. The dataset consists of over 6 M reads of 75 bp in length and is over 900 MB in size. Using this laptop, which was equipped with a dual-core 1.3 GHz Intel i5 processor, WGSQuikr completed analyzing the mock community in less than 8 minutes and used no more than 2 GB of RAM. In contrast, using a much more powerful hexa-core 2.66 GHz Intel Xeon X5650, MetaPhyler took 5.5 hours and MetaPhlAn took 2.9 hours.

**Figure 5:**
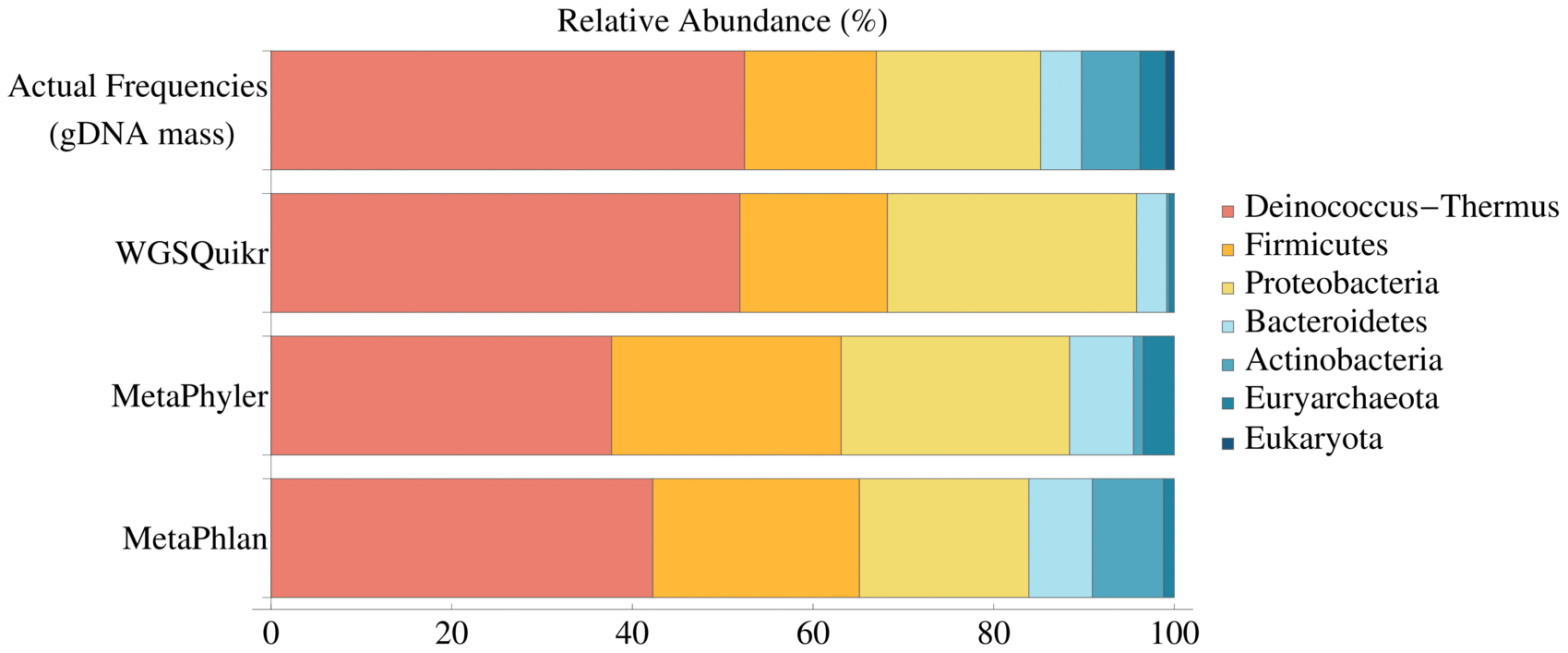
Relative abundances at the phylum level for reconstructions of organisms in the mock community.

The relative abundances of the organisms in the mock community are shown in figure 5 along with their predicted abundance for all three methods. Eukaryota where not included in the training databases of any of the methods, hence its absence in figure 5.

As figure 5 indicates, out of all three methods, WGSQuikr recreates the relative abundance of the most frequently occurring phyla most accurately, at the expense of less accurate abundance estimation of the more rare phyla. This behavior was also observed at the genus level. This indicates that WGSQuikr is an effective tool for rapidly determining the predominant structure of a given metagenomic sample, at the expense of less accurate reconstruction of rare taxa.

### 3.10. Cross Validation

To gauge how well the WGSQuikr method will perform when the given sample contains bacteria not in the database (simulating novelty), we performed a 10-fold cross-validation. Throughout the cross-validation, the 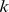-mer size was fixed at 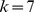. The database 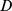 was partitioned into 10 disjoint sets and 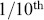 was set aside as testing data with the remaining 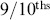 used to form a new 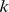-mer matrix. Grinder [9] parameters were then chosen to generate a test sample from the testing data. In particular, these parameters were chosen as follows: read lengths normally distributed with a mean of 150 bp and a standard deviation of 5 bp, 1 M total reads, a power law abundance model, a diversity of 10 species, and the homopolymer error model as in [19]. The mean 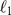-error was then taken over the choice of which 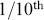 was the testing data. Lastly, an average was taken over 100 iterates of this procedure.

Table 2 summarizes the results of this procedure. The small mean and variance indicates that WGSQuikr performs well at the phylum level, even if a significant portion (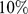) of the sample contains sequences not present in the database. At the genus level, the reconstruction was less accurate (compare to figure 3), indicating that WGSQuikr will benefit from the inclusion of as many bacterial genomes as possible. Hence, WGSQuikr performs best at a taxonomic rank that minimizes the number of novel taxa.

**Table 2:** Results of 100 iterates of the 10-fold cross-validation procedure for WGSQuikr at the phylum and genus levels.

Taxonomic Rank	Mean 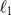 -error 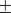 variance
Phylum	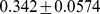
Genus	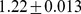

## Conclusion

WGSQuikr represents a new class of metagenomics algorithms, one in which the taxonomic assignments of an entire WGS metagenome are computed, instead of performing the assignment in a read-by-read fashion. This allows for nearly constant execution time and low memory usage, and so is particularly well suited for analyzing very large datasets on a standard laptop computer. In contrast to current methods such as MetaPhyler and MetaPhlAn, WGSQuikr can be used to analyze metagenomes consisting of very short reads (such as the 35-50 bp datasets generated by Illumina's MiSeq) in less than a few hours. As Illumina's advertised quality scores for such short read datasets are typically much higher than for longer read datasets, this may allow for more accurate analysis of metagenomes in unprecedentedly short time frames.

## Supporting Information

Appendix S1 (PDF)
