# Supplementary material for: WGSQuikr: Fast Whole-Genome Shotgun Metagenomic Classification
Source: PLoS One. 2014 Mar 13;9(3):e91784. doi: 10.1371/journal.pone.0091784 (PMC3953531; doi:10.1371/journal.pone.0091784)
Supplement: Appendix S1 — (PDF) [file pone.0091784.s001.pdf]

Section S1: Design of Grinder Experiments

We detail here the production of the simulated data produced by the shotgun/amplicon read simulator Grinder [1]. These datasets were designed to mimic reads generated by a variety of Illumina platforms, and hence we set the read-length distributions to be normally distributed with a variety of means which are summarized in table S1. An equal number of datasets were generated consisting of the following

Table S1: Grinder experiment read lengths.

Mean (bp)	Standard Deviation (bp)	Number of Experiments
35	0	96
50	0	96
100	0	96
150	5	96
250	20	96
300	25	96
450	50	96
800	100	96

number of reads: 10K, 100K, 1M, 5M. Three different diversity values were chosen to be 10, 20, and 50 with abundances modeled by using the following four distributions: linear, uniform, power-law with parameter 0.750 and exponential with parameter 1. Homopolymers of length n were generated by a normal distribution with mean n and variance $0.15 * \sqrt{n}$ as in [2]. Sequencing errors were designed to model Illumina errors and used the 4th degree polynomial in [3] with 80% of these errors set to be substitutions, while the remaining 20% set to be indels. Reference sequences were sampled proportionally to their length to mimic the length bias seen in WGS datasets.

Section S2: Quikr Method Technical Details

Mathematical Formulation. Given the alphabet $\mathcal{A} = \{A, C, T, G\}$, let $\mathcal{A}^n$ denote the set of all words v of length $|v| = n$ on $\mathcal{A}$, and let $\mathcal{A}^* = \bigcup_{n \geq 0} \mathcal{A}^n$ be the set of all finite words on $\mathcal{A}$. Hence words containing non-$ACTG$ characters are ignored. Let $D = \{d_1, \dots, d_M\}$ be a database of genomic sequences $d_j \in \mathcal{A}^*$ and let $S = \{s_1, \dots, s_t\}$ be a set of sample sequences (the reads to be classified). Fix a k-mer size and endow $\mathcal{A}^k = \{v_1, \dots, v_{4^k}\}$ with the lexicographic order. Let $\mathrm{occ}_v(w)$ represent the number of occurrences (with overlap) of the subword v in the word w. That is, for $w, v \in \mathcal{A}^n$, let

(A.1) $$\mathrm{occ}_v(w) = |\{j : w_j w_{j+1} \cdots w_{j+|v|-1} = v\}|.$$

For $j = 1, \dots, M$ and $i = 1, \dots, 4^k$, define the *k-mer training matrix* entrywise as

(A.2) $$A^{(k)}_{i,j} = \frac{occ_{v_i}(d_j)}{|d_j| - k + 1}.$$

The matrix $A^{(k)}$ satisfies $A^{(k)}_{i,j} \geq 0$ and is column-normalized, i.e.

(A.3) $$\sum_{i=1}^{4^k} A^{(k)}_{i,j} = 1 \quad \text{for all } j = 1, \dots, M.$$

Define the *sample k-mer frequency vector* entrywise for $i = 1, \dots, 4^k$ as

(A.4) $$s^{(k)}_i = \frac{\sum_{j=1}^{t} occ_{v_i}(s_j)}{\sum_{l=1}^{4^k} \sum_{j=1}^{t} occ_{v_l}(s_j)}.$$

We assume even coverage of each genome. That is, we assume that the composition of the bacterial community is represented by a probability vector $x \in \mathbb{R}^M$ satisfying the following: given a database sequence $d \in D$, the set of reads $\{s_1^d, \ldots, s_{t_d}^d\} \subset S$ coming from this sequence, and x_d the concentration in the sample of the bacteria corresponding to sequence d, for each i the following holds:

$$\text{(A.5)} \qquad \frac{\sum_{j=1}^{t_d} occ_{v_i}(s_j^d)}{\sum_{l=1}^{4^k} \sum_{j=1}^{t_d} occ_{v_l}(s_j^d)} = x_d \times \frac{occ_{v_i}(d)}{\sum_{l=1}^{4^k} occ_{v_l}(d)}.$$

This means that the total k-mer count of all the read fragments corresponding to the sequence d is proportional to the k-mer count of the sequence d itself, with the proportionality constant being equal to the concentration of the sequence d in the sample S. Our assumptions imply that

$$\text{(A.6)} \qquad A^{(k)}x = s^{(k)}.$$

We will try to recover the probability vector x satisfying $x_j \geq 0$ for all $j = 1, \ldots, M$ and $\sum_{j=1}^{M} x_j = 1$ from information in the form of equation (A.6).

Nonnegative Basis Pursuit Denoising. Given that a bacterial community is typically distributed as a sparse vector x (a small percentage of all extant bacteria are actually present in a given sample), we pursue sparsity-promoting minimizations involving the ℓ_1-norm. Basis Pursuit [4], called (BP) below, is one of the most popular methods. In our situation, it is natural to include the nonnegativity constraint, leading to ($\text{BP}_{\geq 0}$). We further modify the optimization by relaxing the equality constraint to arrive at the regularized problem (REG_1^2). Thus, the three optimization problems considered are:

$$\text{(BP)} \qquad \underset{z \in \mathbb{R}^M}{\text{minimize}} \; ||z||_1 \qquad \text{subject to } A^{(k)}z = s^{(k)},$$

$$(\text{BP}_{\geq 0}) \qquad \underset{z \in \mathbb{R}^M}{\text{minimize}} \; ||z||_1 \qquad \text{subject to } A^{(k)}z = s^{(k)} \text{ and } z \geq 0,$$

$$(\text{REG}_1^2) \qquad \underset{z \in \mathbb{R}^M}{\text{minimize}} \; ||z||_1^2 + \lambda^2 ||A^{(k)}z - s^{(k)}||_2^2 \qquad \text{subject to } z \geq 0,$$

It can be demonstrated, thanks to (A.3), that (BP) and ($\text{BP}_{\geq 0}$) are equivalent in the sense that x is a solution of (BP) if and only if it is a solution of ($\text{BP}_{\geq 0}$), and that the latter is approached by solutions of (REG_1^2) when $\lambda \to \infty$, see [4].

We shall solve (REG_1^2) since it has the notable advantage of being transformed into a nonnegative least squares problem. Indeed, with

$$\text{(A.7)} \qquad \tilde{A}^{(k)} := \left[\frac{1 \cdots 1}{\lambda A^{(k)}} \right], \qquad \tilde{s}^{(k)} := \left[\frac{0}{\lambda s^{(k)}} \right],$$

the minimization (REG_1^2) is equivalent to

$$\text{(NNLSQ)} \qquad \underset{z \in \mathbb{R}^M}{\text{minimize}} \; ||\tilde{A}^{(k)}z - \tilde{s}^{(k)}||_2^2 \qquad \text{subject to } z \geq 0.$$

Algorithmic Implementation. To solve (NNLSQ) we utilized MATLAB's [5] implementation of `lsqnonneg()` which in turn is an implementation of the iterative Lawson-Hanson algorithm described in [6]. To calculate the matrices $A^{(k)}$ and the vector $s^{(k)}$ we used a custom SML [7] subword counting program written by Christopher Cramer and compiled for Linux using MLton [8].

Selection of λ. Parameter tuning is a common issue to be addressed when using regularized optimization procedures to solve linear inverse problems [9–11]. Two common methods include Generalized Cross Validation [12] and the L-Curve method [13]. The adaptive method by which we select the λ used in (A.7) is similar in spirit to the L-Curve method. In every case, it was observed that as a function of λ, the number of iterates necessary to solve (NNLSQ) via the Lawson-Hanson algorithm was linear, then exponential, then non-increasing. Figure S1 demonstrates this phenomenon for a sample dataset by plotting the number of iterates as a function of λ. Let $its(\lambda)$ be the number of iterates needed to

solve (NNLSQ) via the Lawson-Hanson algorithm. It was also observed that x was most accurately reconstructed at the λ for which the number of iterations experienced its greatest increase. Figure S2 demonstrates this fact for an example dataset by plotting the ℓ_1-error as well as $\frac{d}{d\lambda}its(\lambda)$ as a function of λ, where $\frac{d}{d\lambda}its(\lambda)$ denotes the first differences of $its(\lambda)$.

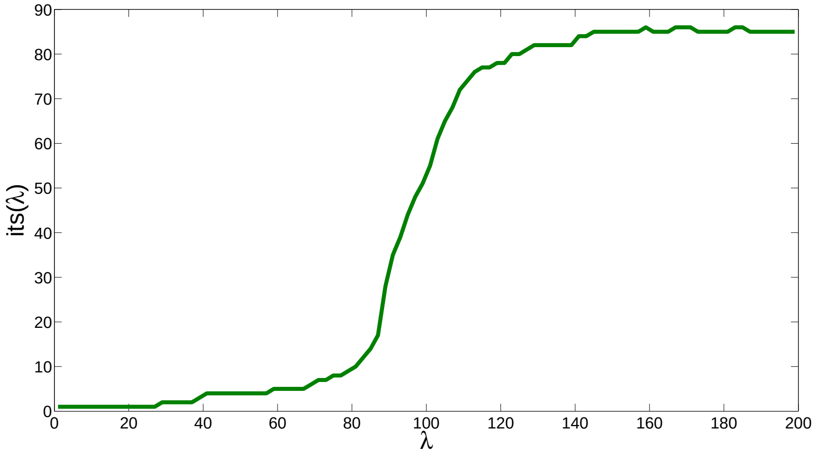

Figure S1: Number of iterates to solve (NNLSQ) as a function of λ.

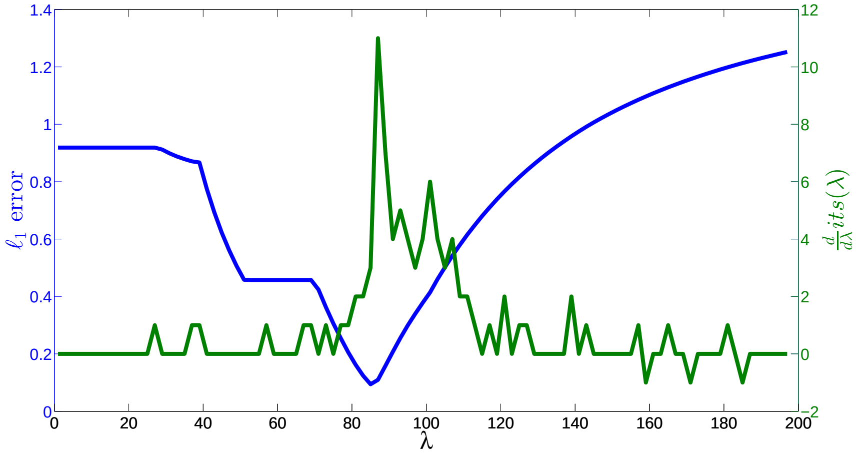

Figure S2: ℓ_1-error and first difference of number of iterates needed to solve (NNLSQ) as functions of λ.

With this information in hand, the following adaptive approach was used to choose λ. First, let $\frac{d}{d\lambda}its(\lambda_{t_0:I:t_1}))$ designate the first differences of $its(\lambda)$ when λ ranges from t_0 to t_1 in increments of I. We allowed λ to increase in increments of 100 from $\lambda = 1$ until a smoothing spline approximation of $\frac{d}{d\lambda}its(\lambda_{1:100:t_1})$ was shown to be negative for some t_1. A smoothing spline approximation was utilized because $\frac{d}{d\lambda}its(\lambda_{t_0:I:t_1})$ is noisy when increasing λ in such large increments. Next, the maximum of $\frac{d}{d\lambda}its(\lambda_{1:100:t_1})$ with respect to λ was identified, call it $\lambda = t_M$. Then the maximizer of

$\frac{d}{d\lambda} its(\lambda_{t_M-100:10:t_M+100})$ was used as the value of λ to solve (NNLSQ). Clearly this method can be refined further by taking smaller and smaller increments surrounding the maximizer of $\frac{d}{d\lambda} its(\lambda)$, but we found this two-step approach to provide sufficient speed and accuracy improvements over current methods.

References

1. Angly FE, Willner D, Rohwer F, Hugenholtz P, Tyson GW (2012) Grinder: a versatile amplicon and shotgun sequence simulator. Nucleic acids research 61: 1–8.
2. Richter DC, Ott F, Auch AF, Schmid R, Huson DH (2008) MetaSim: a sequencing simulator for genomics and metagenomics. PloS ONE 3: e3373.
3. Korbel JO, Abyzov A, Mu XJ, Carriero N, Cayting P, et al. (2009) PEMer: a computational framework with simulation-based error models for inferring genomic structural variants from massive paired-end sequencing data. Genome biology 10: R23.
4. Foucart S, Koslicki D (2013) Sparse Recovery by means of Nonnegative Least Squares. IEEE Signal Processing Letters, In Print .
5. MATLAB 2012b, The MathWorks, Inc., Natick, MA, USA.
6. Lawson C, Hanson R (1974) Solving Least Squares Problems. Prentice-Hall, 350 pp.
7. Milner R, Tofte M, Harper R (1997) The Definition of Standard ML. Cambridge, MA: MIT press, 128 pp.
8. Weeks S (2006) Whole-program compilation in MLton. In: Proceedings of the 2006 workshop on ML. New York, NY.: ACM, p. 1.
9. Vogel C (2002) Computational Methods for Inverse Problems. Philadelphia, PA: SIAM, 183 pp.
10. Bertero M, Boccacci P (1998) Introduction to Inverse Problems in Imaging. London: Institute of Physics Publishing, 352 pp.
11. Daubechies I, Defrise M, De Mol C (2004) An iterative thresholding algorithm for linear inverse problems with a sparsity constraint. Comm Pure Appl Math 57: 1413–1457.
12. Golub G, Heath M, Wahba G (1979) Generalized cross-validation as a method for choosing a good ridge parameter. Technometrics 21: 215–223.
13. Hansen P, O'Leary D (1993) The use of the L-curve in the regularization of discrete ill-posed problems. SIAM J Sci Comput 14: 1487–1503.
